# Temperature effects on the calculation of the functional derivative of T_c_ with respect to *α*^2^*F*(*ω*)

**DOI:** 10.1371/journal.pone.0286855

**Published:** 2023-06-06

**Authors:** J.A. Camargo-Martínez, F. Mesa, G.I. González-Pedreros

**Affiliations:** 1 Grupo de Investigación en Ciencias Básicas, Aplicación e innovación- CIBAIN, Unitrópico, Yopal, Colombia; 2 Fundación Universitaria Los Libertadores, Facultad de Ingeniería y Ciencias Básicas, Bogotá, Colombia; 3 Universidad Pedagógica y Tecnológica de Colombia, Facultad de Ciencias, Tunja, Colombia; Tel Aviv University, ISRAEL

## Abstract

The functional derivative of the superconducting transition temperature T_c_ with respect to the electron-phonon coupling function $\alpha^{2}F(\omega ),\delta T_{c}^{2}/\delta \alpha^{2}F(\omega )$ permits identifying the frequency regions where phonons are most effective in raising T_c_. This work presents an analysis of temperature effects on the calculation of the *δT*_*c*_/*δα*^2^*F*(*ω*) and μ* parameters. The results may permit establishing that the variation of the temperature in the *δT*_*c*_/*δα*^2^*F*(*ω*) and μ* parameter allows establishing patterns and conditions that are possibly related to the physical conditions in the superconducting state, with implications on the theoretical estimation of the T_c_.

## Introduction

Superconductivity is the complete loss of electrical resistivity of a material that occurs only below a certain temperature, called superconducting critical temperature T_c_. It is a state of matter with technologically impactful applications but with serious difficulties of use on a large scale due to the extreme conditions in which it occurs: low temperatures or high pressures. However, its application on a small scale is a current fact.

Research on the subject from a theoretical approach seeks to establish its fundamental physical mechanisms, the understanding of which will positively lead to the engineering of superconducting materials with a view to their application on a large scale. The best approach, recognized with the Nobel Prize in physics in 1971, is the Bardeen-Cooper-Schrieffer (BCS) theory, which states that superconductivity is the “physics of Cooper pairs” [1].

**Fig 1:**
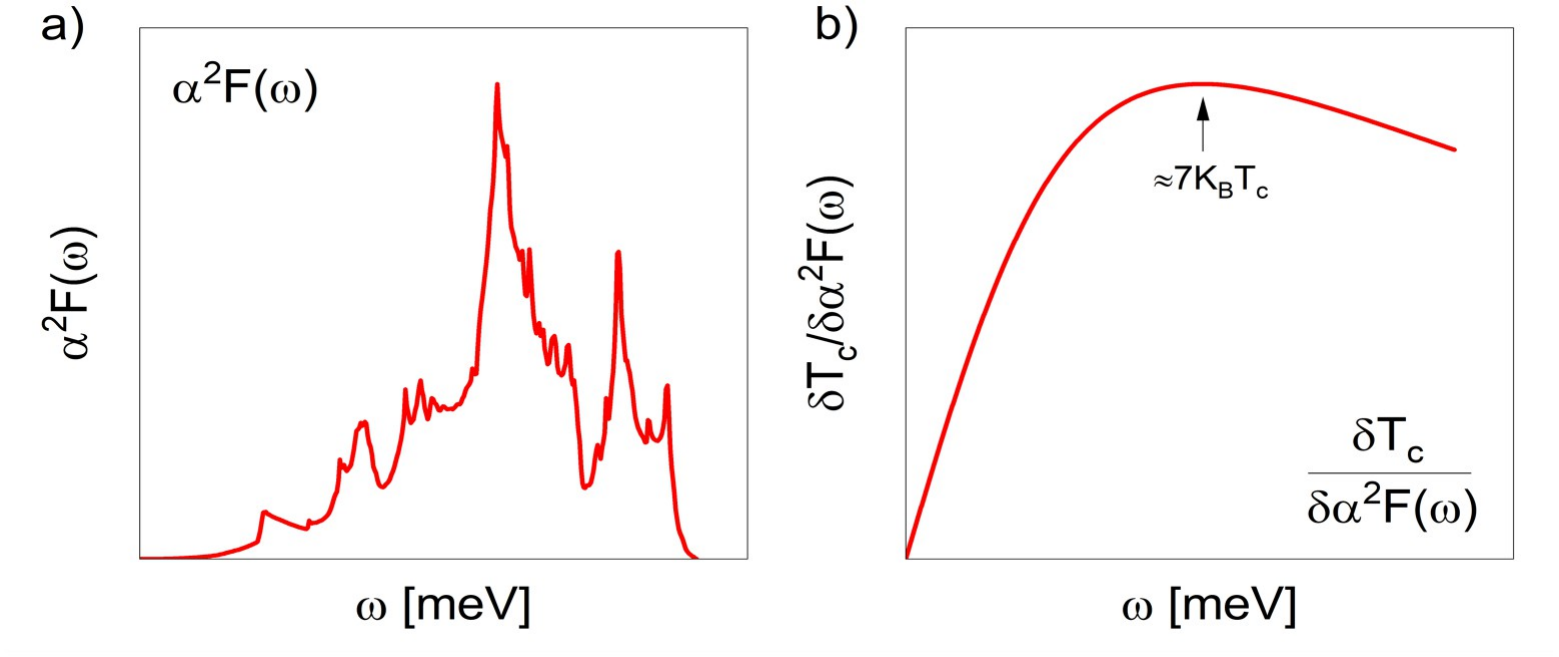
Schematics of (a) the Eliashberg spectral function *α*^2^*F*(*ω*) and (b) the functional derivative of the superconducting critical temperature *T*_*c*_ concerning the *α*^2^*F*(*ω*) function, Δ*T*_*c*_/*δα*^2^*F*(*ω*).

Here, the effective attraction between electrons forming the Cooper pair is generated by the interaction between the electrons and the lattice vibrations (phonons), called the electron-phonon interaction. This scheme explains the phenomenon for weak electron-phonon coupling systems, leading to T_c_ below 70 K, (lower than the temperature of liquid nitrogen). Thus, the next step was to generalize the BCS theory to superconductors, in which the electron-phonon interaction is strong and hence has a higher T_c_ This was the work of G. M. Eliashberg [2] who in his theoretical description, introduced the electron-phonon interaction and the electronic and phononic band structure more precisely. All that information is gathered in a function, the Eliashberg spectral function, or electron-phonon coupling function *α*^2^*F*(*ω*) (see Fig 1), which can be obtained both theoretically (DFT calculations) and experimentally (tunneling experiment). The Eliashberg spectral function is obtained from the calculated phonon spectrum and the calculated electron–phonon matrix elements [3, 4]. The Coulombic repulsion between electrons is included through a parameter *μ*.

On the other hand, the linearization of the Eliashberg equations makes it possible to determine the functional derivative of the superconducting critical temperature T_c_ concerning the function *α*^2^*F*(*ω*), *δF*_*c*_/*δα*^2^*F*(*ω*). The first numerical calculations of the *δF*_*c*_/*δα*^2^*F*(*ω*) in superconductors were performed by Bergmann and Rainer [5]. Their results showed that this function has a universal form (see Fig 1b): it grows from *ω* = 0 to a maximum at *ω* ∼ 7*K*_*B*_*T*_*c*_ and then slowly decreases to 0 as *ω* → ∞ [5, 6]. From *δT*_*c*_/*δα*^2^*F*(*ω*), it is possible to determine the phonon frequency leading to the highest possible T_c_ in a superconductor [7] and to describe the change in T_c_, Δ*T*_*c*_, given a slight variation in the *α*^2^*F*(*ω*) function, Δ*α*^2^*F*(*ω*), generated by the action of physical conditions such as pressure, doping, etc. [8–10], thus (Eq (1);

$$\begin{array}{c} \Delta T=\int_{0}^{+\infty }\frac{\Delta T_{c}}{\delta \alpha^{2}F\left(\omega \right)}\Delta \alpha^{2}F\left(\omega \right)d\omega \end{array}$$ (1)

A previous theoretical study showed that there is a correlation between the frequencies of the maxima of the *δT*_*c*_*δα*^2^*F*(*ω*) and *α*^2^*F*(*ω*) functions [11, 12], where the convergence of these frequencies occurs at the optimal electron-phonon interaction conditions leading to the superconductor reaching the maximum possible $T_{c}(T_{c}^{Max})$ [13–17]. This convergence frequency is called the optimum frequency *ω*_*opt*_, which satisfies the relation $\omega_{opt}=7K_{B}T_{c}^{Max}$, where *K*_*B*_ is the Boltzmann constant. The calculation of the *δT*_*c*_/*δα*^2^*F*(*ω*) requires the experimental (or test) *T*_*c*_ value for the prior determination of the parameter μ*, which physically accounts for the Coulombic repulsion between electrons in the system under study. The parameter μ* is determined from the fit to the linearized Eliashberg equations (see Materials and methods section- Eq (3) when the pair breakdown parameter *ρ* tends to zero (*ρ* → 0), which is valid for *T* = *T*_*c*_ [15].

Up until now, the physical interpretation and application of the *δT*_*c*_/*δα*^2^*F*(*ω*) have failed to consolidate. In 2015 Nicol and Carbotte used the *δT*_*c*_/*δα*^2^*F*(*ω*) to demonstrate that the *α*^2^*F*(*ω*) spectral function of sulfur trihydride H_3_S at 200 GPa is highly optimized for *T*_*c*_ [18]. González-Pedreros and Baquero [10] and Camargo-Martínez *et al.* [19] used the *δT*_*c*_/*δα*^2^*F*(*ω*) to determine the trend of *T*_*c*_ as a function of pressure in Nb-bcc (Cubic Niobium) and H_3_S respectively, taking the reported experimental *T*_*c*_ as a starting point. In other work, the *δT*_*c*_/*δα*^2^*F*(*ω*) was determined to identify possible frequency regions where phonons would be the most effective in increasing *T*_*c*_ [20, 21]. All these results are descriptive and not predictive in nature.

One of the possible contributions of theoretical physics in superconductivity is to clearly establish the fundamental physical foundations of the superconducting phenomenon in order to suggest with certainty, the line of experimental process to obtain superconductivity at room temperature in viable conditions for its application to large-scale. An example of the predictive effect of the theoretical approach on superconductivity was observed in the idea proposed by Ashcroft [22], who stated that hydrogen-rich systems would be viable candidates to be high critical temperature superconductors. This proposal gave rise to experimentation in this field with the discovery of new high-Tc superconductors, as H_3_S (*T*_*c*_ of 203 K at 155 GPa [23]) or LaH_10_ (*T*_*c*_ of 260 K at 180 GPa [24]), called hydride superconductors. This discovery gave a new impetus to this field of study, which had been stuck with the superconducting cuprates (*T*_*c*_ of 164 K) since 1994 [25]. The current difficulty with hydride superconductors is in their high-pressure conditions of formation. In this sense, evaluating possible new ways to predict *T*_*c*_ values in terms of well-defined physical conditions (such as pressure, doping, etc.) is an interesting line of work. Here, the study of the functional derivative *δT*_*c*_/*δα*^2^*F*(*ω*) seeks to establish the possible existence of patterns that lead to the determination of an optimum temperature of the system (superconducting critical temperature), which would also avoid the use of test or experimental *T*_*c*_ in first-principles calculations.

From a purely computational point of view, the temperature value can have pivotal implications in the calculation, result, and interpretation of the functional derivative *δT*_*c*_/*δα*^2^*F*(*ω*). For this reason, in this manuscript, we present the analysis of the effects of temperature variation, around experimental *T*_*c*_ value, on the calculation of the functional derivative *δT*_*c*_/*δα*^2^*F*(*ω*), in the superconductor H_3_S, of which a *T*_*c*_ of 203 K at 155 GPa was measured [23].

## Materials and methods

This study was developed based on the Eliashberg *α*^2^*F*(*ω*) spectral functions of H_3_S obtained in previous work [12, 19], whose calculations were performed in the range of pressures (155–225 GPa), where the experimental *T*_*c*_ were reported [23]. Here, the functional derivatives were obtained with the procedure widely used by Carbotte *et al.* [13–16, 18, 26, 27] which is based on the work of G. Bergmann and D. Rainer [5]. The determination of the functional derivative (see Eq (2)) of the superconducting critical temperature *T*_*c*_ with respect *α*^2^*F*(*ω*) function, *δT*_*c*_/*δα*^2^*F*(*ω*), was performed from the relation:

$$\begin{array}{c} \frac{\delta T_{c}}{\delta \alpha^{2}F\left(\omega \right)}=\frac{\frac{\delta \rho }{\delta \alpha^{2}F\left(\omega \right)}}{{\left(\frac{\partial \rho }{\partial T}\right)}_{T_{c}}} \end{array}$$ (2)

Where *ρ* was expressed in terms of *K*_*nm*_ for *T* = *T*_*c*_, which is obtained as a (kernel) solution of the linearized Eliashberg equations on the imaginary axis [5, 6]:

$$\begin{array}{c} \rho {\bar{\Delta }}_{n}=\pi T\sum_{m}(\lambda_{nm}-\mu^{*}-\delta_{nm}\frac{|\tilde{\omega }|}{\pi T}){\bar{\Delta }}_{m} \end{array}$$ (3)

with $\tilde{\omega }=i\omega_{n}+\pi T\sum_{m}\lambda_{nm}sgn(\omega_{n}$), *ω*_*n*_ = *πT*(2*n* − 1) the n-th Matsubara frequency and ${\bar{\Delta }}_{n}=\frac{\tilde{\Delta_{n}}}{\rho +|\tilde{\omega_{n}}|}$. For more details on the mathematical formulation, see the reference [17]. To evaluate the effects of the *T*_*c*_ parameter on the *δT*_*c*_/*δα*^2^*F*(*ω*) calculations, 10 K variations in temperature around the experimental value (reference temperature) were developed for each of the pressures evaluated (155 GPa,175 GPa, 195 GPa, and 215 GPa).

## Results and discussion

**Fig 2:**
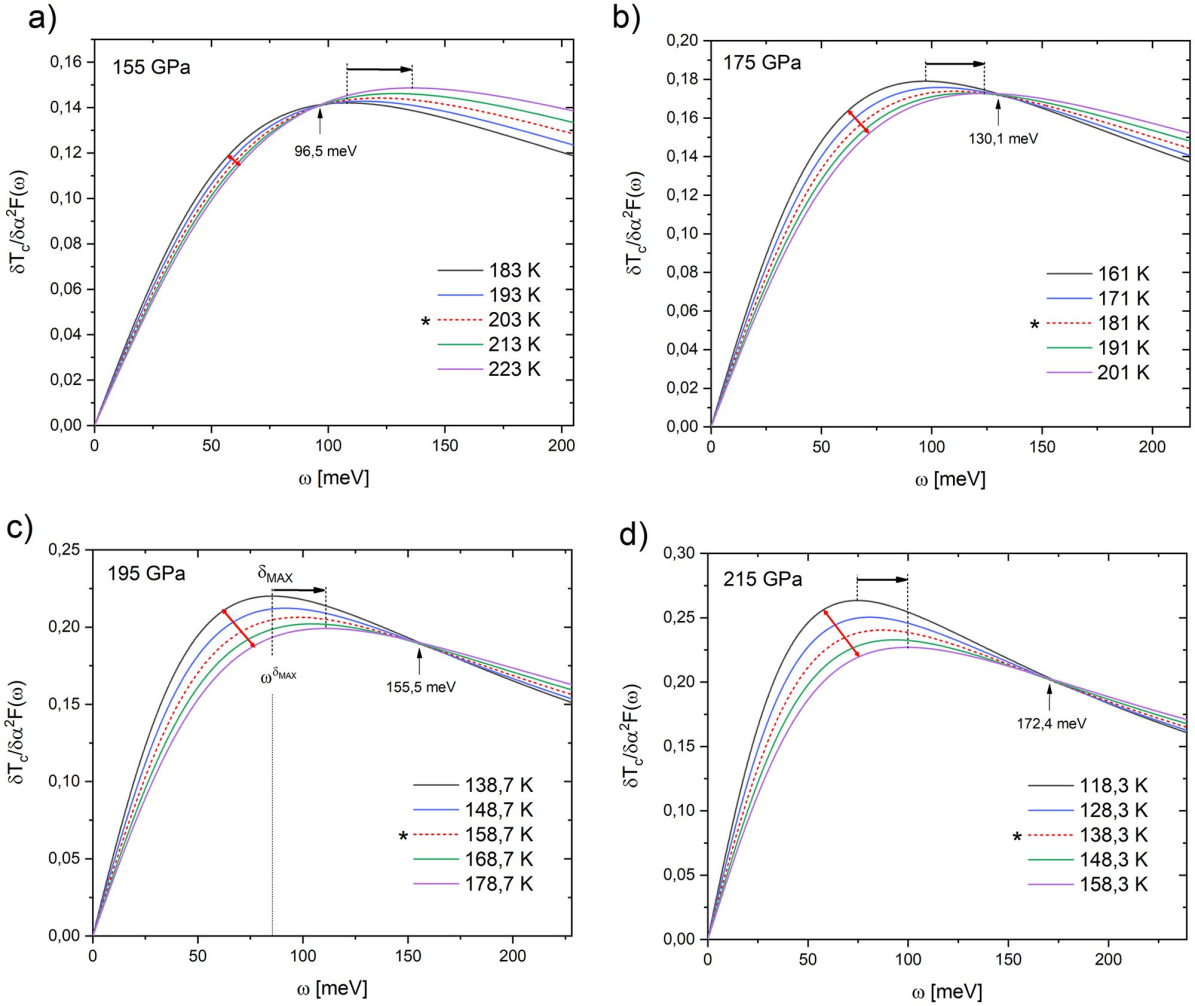
*δT*_*c*_/*δα*^2^*F*(*ω*) as a function of frequency *ω* for H_3_S calculated at different temperatures at pressures of a) 155GPa, b) 175 GPa, c) 195 GPa, and d) 215 GPa. Stars show critical temperature related to corresponding pressure, horizontal arrows indicate frequency shift of the maximum value of the *δT*_*c*_/*δα*^2^*F*(*ω*), $\omega^{\delta_{MAX}}$, red arrows present how *δT*_*c*_/*δα*^2^*F*(*ω*)-curve increases their separation, and vertical arrows point average frequency of intersection of the *δT*_*c*_/*δα*^2^*F*(*ω*).

The *δT*_*c*_/*δα*^2^*F*(*ω*) as a function of frequency calculated for H_3_S at different pressures and temperatures are presented in Fig 2.

Each case evaluated (Fig 2), frequency values in every *δT*_*c*_/*δαT*2*F*(*ω*)-maximum are displaced, *δ*_*MAX*_, as temperature is increased in accord with relation *ω*_*opt*_ ∼ *k*_*B*_*T*_*c*_ [5]. The *δT*_*c*_/*δα*^2^*F*(*ω*)-maximum allows identifying of the frequency regions *ω*_*opt*_ where phonons are more effective in increasing *T*_*c*_ [28]; this is why it is so important to evaluate their behavior.

To every pressure (Fig 2a–2d), in a temperature interval Δ*T* = 40*K* about *T*_*c*_, *δT*_*c*_/*δα*^2^*F*(*ω*) intersects a narrow frequency range (it looks point-like) is verified. Condition of small variation of the *δT*_*c*_/*δα*^2^*F*(*ω*) due to temperature effects, below the intersection point, is observed for the system under the lowest compression (155 GPa) and vice versa. This behavior is opposite if the *δT*_*c*_/*δα*^2^*F*(*ω*) are compared at frequencies higher than the intersection point. In both cases, the effect of temperature on the calculation of the *δT*_*c*_/*δα*^2^*F*(*ω*) could establish patterns (intersection point, variation, or separation between the *δT*_*c*_/*δα*^2^*F*(*ω*) and their maxima) that would lead to the determination of optimal physical conditions of the superconducting state and the possible estimation of the *T*_*c*_.

Now, the intensities (value on the vertical axis) of the *δT*_*c*_/*δα*^2^*F*(*ω*)-maximums show two different behaviors (Fig 2). At 155 GPa, such intensities slightly increase their value as the temperature increases, as a consequence of the little separation induced in the *δT*_*c*_/*δα*^2^*F*(*ω*). However, for the other pressures (175, 195, and 215 GPa), the behavior of the *δT*_*c*_/*δα*^2^*F*(*ω*) intensities is opposite to the 155 GPa case, starting from a higher intensity and decreasing with increasing temperature, being more evident with increasing pressure. It is important to note that there seems to be no relationship between the variation of the frequency of the maximum of the *δT*_*c*_/*δα*^2^*F*(*ω*),$\omega^{\delta_{MAX}}$, and the intensity of the maximum of the *δT*_*c*_/*δα*^2^*F*(*ω*).

**Fig 3:**
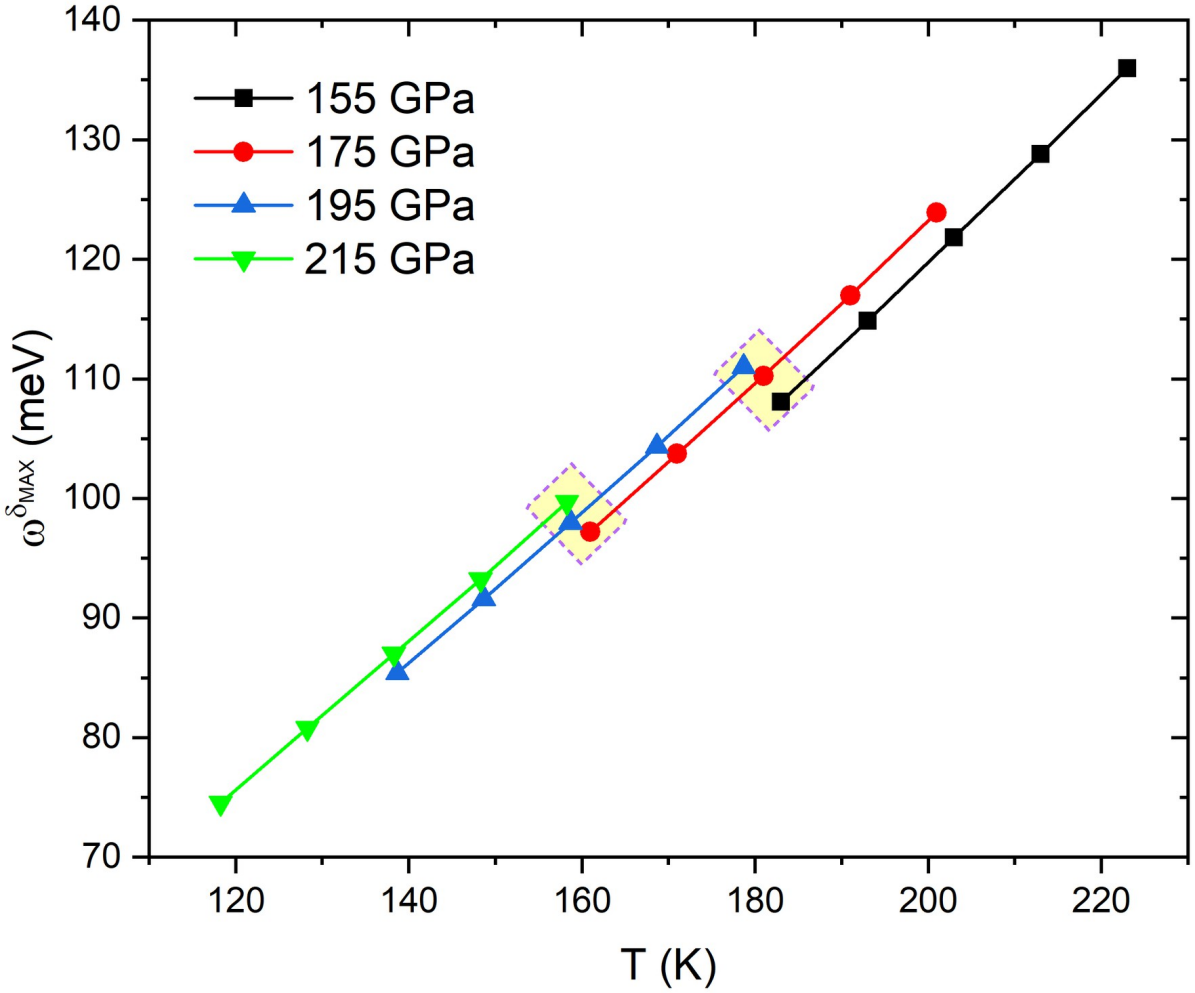
Frequency of the maximum of the *δT*_*c*_/*δα*^2^*F*(*ω*),$\omega^{\delta_{MAX}}$, as a function of temperature, at different pressures. The dashed boxes (yellow) show the slight change in $\omega^{\delta_{MAX}}$ induced by pressure.

The patterns of the *δT*_*c*_/*δα*^2^*F*(*ω*) vs *T*_*c*_ in the H_3_S reveal that these seem to have a characteristic behavior at a specific pressure (155 GPa).

(Fig 3) shows the linearity of the frequency of the maximum of the $\delta T_{c}/\delta \alpha^{2}F(\omega )(\omega^{\delta_{MAX}})$ as a function of temperature for all pressures. Such lines are collinear with mean slope $\bar{m}=+$0.64 meV/K. This means that the $\omega^{\delta_{MAX}}$ moves uniformly toward higher frequencies as the temperature increases. On the other hand, $\omega^{\delta_{MAX}}$ is almost unaffected by the pressure (p) since a considerable change of Δ*p* = 40 GPa induces a small $\Delta \omega^{\delta_{MAX}}=2.5$ meV. However, each pressure has a limit of $\omega^{\delta_{MAX}}$, whose maximum value is reached at 155 GPa, leading to a higher *T*_*c*_.

**Fig 4:**
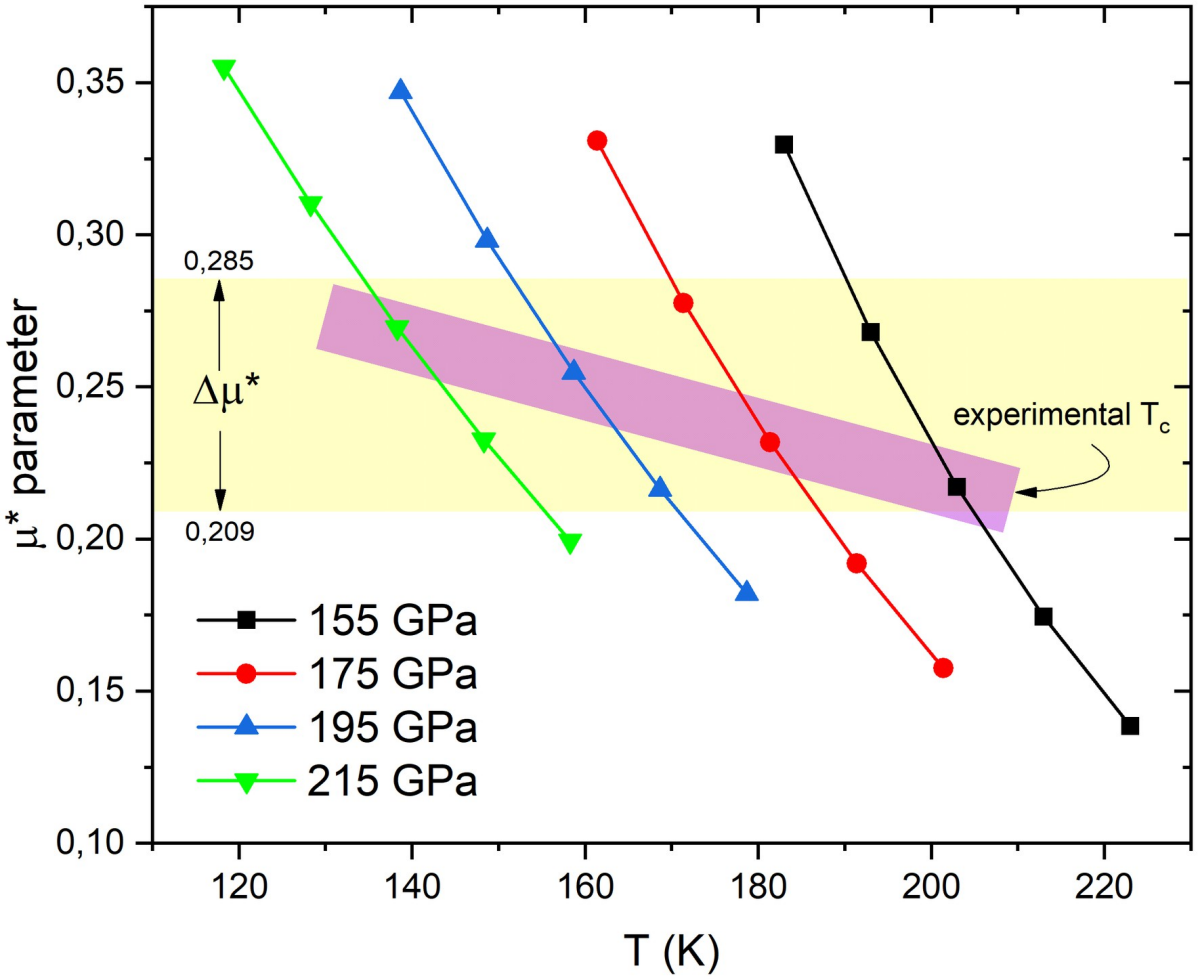
The parameter μ* as a function of temperature T for H_3_S at different pressures. The horizontal band (yellow) marks the Δμ* within which the μ* fitted to the experimental *T*_*c*_ are set, indicated by the diagonal band (purple).

In the calculation of the *δT*_*c*_/*δα*^2^*F*(*ω*), the temperature variation involves the determination of the μ* parameter of Fig 3. The comparison between the parameters μ* adjusted at different temperatures for each of the pressures is presented in Fig 4.

It is observed in Fig 4 that μ* vs T has a comparable trend between pressures. This could be assumed to be quasi-linear in a first approximation. However, this quasi-linearity is much more evident at higher pressure. The results show that a Δ*T*_*c*_ = 40*K* induces a $\bar{\Delta \mu^{*}}=0.25$. However, the μ* values fitted to the experimental *T*_*c*_ in the pressure range from 155 to 215 GPa are in the Δμ* range of 0.209–0.285, which implies a $\bar{\Delta T_{c}}=18.1$ K, a small range with respect to the 203 K of the experimental maximum *T*_*c*_ of H_3_S. This result is interesting because it would allow establishing an initial criterion of theoretical estimation of the *T*_*c*_ around a small range of temperatures, according to the Δμ*. It is then necessary to determine, with this procedure, the Δμ* other systems to evaluate if this presents a universal range or if it varies significantly from one system to another.

In the calculation of the *δT*_*c*_/*δα*^2^*F*(*ω*), it was found that for temperatures distant between -60 K and +30 K with respect to the experimental *T*_*c*_, μ* values of 0,8 and 0,09 are generated, which are outside the values typically used or calculated (between 0,3 and 0,1), and their *δT*_*c*_/*δα*^2^*F*(*ω*) presented computational difficulties in their calculation, with behaviors different in form from those observed in the *δT*_*c*_/*δα*^2^*F*(*ω*) calculated at temperatures close to the experimental *T*_*c*_.

## Conclusions

This paper presents the preliminary theoretical analysis of the effects of temperature variation around the experimental superconducting critical temperature *T*_*c*_ on the calculation of the functional derivative *δT*_*c*_/*δα*^2^*F*(*ω*) for superconducting H_3_S in the pressure range from 155 to 215 GPa. These calculations included the determination of the μ* parameters through fitting *T*_*c*_ in the linearized Eliashberg equations. The calculated *δT*_*c*_/*δα*^2^*F*(*ω*) revealed temperature- and pressure-induced displacement, intersection, and separation patterns that could be associated with the physical conditions in the superconducting state and the estimation of *T*_*c*_. The μ* values obtained allowed the determination of a range of values leading to temperatures that could establish an initial criterion for possible theoretical estimation of *T*_*c*_. This procedure must be evaluated and confirmed in other similar systems to establish the possible generalization of the results presented here.

## References

[1] Bardeen J, Cooper LN, Schrieffer JR. Theory of Superconductivity. Phys. Rev. 1957;108:1175–1204. doi: 10.1103/PhysRev.108.1175

[2] Eliashberg GM. Interactions between electrons and lattice vibrations in a superconductor. Sov. Phys.—JETP (Engl Transl). 1960;11:3.

[3] Allen PB, Dynes RC. Transition temperature of strong-coupled superconductors reanalyzed. Phys. Rev. B 1975;12:905. doi: 10.1103/PhysRevB.12.905

[4] Karaca E, Arslan E, Tütüncü HM, Srivastava GP. Physical properties of the body-centred tetragonal CaPd_2_Ge_2_. Philosophical Magazine 2017;97(22):1866. doi: 10.1080/14786435.2017.1320439

[5] Bergmann G, Rainer D. The sensitivity of the transition temperature to changes in *α*^2^ *F*(*ω*). Z Physik. 1973;263:59–68. doi: 10.1007/BF02351862

[6] Rainer D, Bergmann G. Temperature dependence of *H*_*c*2_ and *κ*_1_ in strong coupling superconductors. J Low Temp Phys. 1974;14:501–519. doi: 10.1007/BF00658876

[7] Baquero R, Gutiérrez-Ibarra J, Meza L, Navarro O, Kihlstrom KE. Eliashberg theory and high-*T*_*c*_ superconductivity. Revista Mexicana de Física 1989;35(3):161–169.

[8] Baquero R, López-Olazagasti E. Superconducting lanthanum under pressure. Phys. Rev. B. 1984;30:5019.

[9] Mitrović B. A new method for calculating the functional derivative *δT*_*c*_/*δα*^2^ *F*(*Ω*): Application to MgB_2_. Int. J Mod. Phys. C. 2002;13:1087–1093. doi: 10.1142/S012918310200384X

[10] González-Pedreros GI, Baquero R. Superconducting critical temperature under pressure. Physica C: Superconductivity and its Applications. 2018;548:132–137.

[11] González-Pedreros GI, Camargo-Martínez JA, Mesa F. Cooper Pairs Distribution function for bcc Niobium under pressure from first-principles. Sci Rep. 2021;11:7646. doi: 10.1038/s41598-021-87028-x 33828157 PMC8027384

[12] González-Pedreros GI, Camargo-Martínez JA, Mesa F. Cooper-pair distribution function *D*_*cp*_(*ω*, *T*_*c*_) for superconducting D_3_S and H_3_S. Sci Rep. 2021;11:22618. doi: 10.1038/s41598-021-02081-w 34799648 PMC8604968

[13] Daams MJ. Anisotropic Superconductors and Elastic Impurity Scattering. Ph.D. thesis. Mc-Master University, Hamilton, Ont. 1977. Available: https://macsphere.mcmaster.ca/handle/11375/12642

[14] Daams JM, Carbotte JP, Baquero R. Critical field and specific heat of superconducting Tl-Pb-Bi alloys. J Low Temp Phys. 1979;35:547–559. doi: 10.1007/BF00117895

[15] Daams JM, Carbotte JP. Thermodynamics of superconducting Nb_3_Sn. Solid State Communications. 1979;29:501–505. doi: 10.1016/0038-1098(79)90794-4

[16] Daams JM, Carbotte JP. Thermodynamics of strong coupling superconductors including the effect of anisotropy. J Low Temp Phys. 1981;43:263–286. doi: 10.1007/BF00116155

[17] Camargo-Martínez JA, González-Pedreros GI, Mesa F. The higher superconducting transition temperature *T*_*c*_ and the functional derivative of *T*_*c*_ with *α*^2^ *F*(*ω*) for electron–phonon superconductors. J Phys: Condens Matter. 2020;32:505901. 10.1088/1361-648X/abb741 32969350

[18] Nicol EJ, Carbotte JP. Comparison of pressurized sulfur hydride with conventional superconductors. Phys Rev. B. 2015;91:220507. doi: 10.1103/PhysRevB.91.220507

[19] Camargo-Martínez J.A., González-Pedreros G.I., Baquero R. High-*T*_*c*_ superconductivity in H_3_S: pressure effects on the superconducting critical temperature and Cooper pair distribution function. Supercond Sci Technol. 2019;32:125013.

[20] Tanaka K, Tse JS, Liu H. Electron-phonon coupling mechanisms for hydrogen-rich metals at high pressure. Phys Rev B. 2017;96:100502. doi: 10.1103/PhysRevB.96.100502

[21] Yao Y, Tse JS, Tanaka K, Marsiglio F, Ma Y. Superconductivity in lithium under high pressure investigated with density functional and Eliashberg theory. Phys Rev B. 2009;79:054524. doi: 10.1103/PhysRevB.79.054524

[22] Ashcroft N.W. Hydrogen Dominant Metallic Alloys: High Temperature Superconductors? Rev. Lett. 92, 187002 (2004). doi: 10.1103/PhysRevLett.92.187002 15169525

[23] Drozdov AP, Eremets MI, Troyan IA, Ksenofontov V, Shylin SI. Conventional superconductivity at 203 kelvin at high pressures in the sulfur hydride system. Nature. 2015;525:73–76. doi: 10.1038/nature14964 26280333

[24] Somayazulu M, Ahart M, Ajay KM, Geballe ZM, Baldini M, Meng Y, et al. Evidence for Superconductivity above 260 K in Lanthanum Superhydride at Megabar Pressures. Phys. Rev. Lett. 2019;122:027001. doi: 10.1103/PhysRevLett.122.027001 30720326

[25] Gao L, Xue YY, Chen F, Xiong Q, Meng RL, Ramirez D, et al. Superconductivity up to 164 K in HgBa_2_Ca_*m*−1_Cu_*m*_O_2*m*+2+*δ*_ (m = 1, 2, and 3) under quasihydrostatic pressures. Phys. Rev. B 1994;50:4260(R). doi: 10.1103/PhysRevB.50.4260 9976724

[26] Daams J.M., Carbotte J.P. Functional derivatives for the critical magnetic field of Pb. Can. J. Phys. 56 (1978) 1248. doi: 10.1139/p78-165

[27] Daams J., Carbotte J.P. Thermodynamic properties of superconducting niobium J. Low Temp. Phys. 40 (1980) 135.

[28] Mitrović B, Carbotte JP. Tunneling in a superconductor with an energy dependent electronic density of states. Solid State Communications. 1981;40:249–252. doi: 10.1016/0038-1098(81)90751-1

